# Molecular characterisation of multidrug-resistant *Mycobacterium tuberculosis* isolates from a high-burden tuberculosis state in Brazil

**DOI:** 10.1017/S0950268819001006

**Published:** 2019-06-13

**Authors:** R. S. Salvato, S. Schiefelbein, R. B. Barcellos, B. M. Praetzel, I. S. Anusca, L. S. Esteves, M. L. Halon, G. Unis, C.F. Dias, S. S. Miranda, I. N. de Almeida, L. J. de Assis Figueredo, E. C. Silva, A. L. Kritski, E. R. Dalla Costa, M. L. R. Rossetti

**Affiliations:** 1 Programa de Pós-graduação em Biologia Celular e Molecular Aplicada à Saúde, Universidade Luterana do Brasil, Canoas, Rio Grande do Sul, Brazil; 2 Centro de Desenvolvimento Científico e Tecnológico (CDCT), Secretaria Estadual da Saúde do Rio Grande do Sul, Porto Alegre, Rio Grande do Sul, Brazil; 3 Universidade Federal de Ciências da Saúde de Porto Alegre, Porto Alegre, Rio Grande do Sul, Brazil; 4 Faculdade de Medicina, Universidade Federal do Rio de Janeiro, Rio de Janeiro (RJ), Brazil; 5 Hospital Sanatório Partenon, Porto Alegre, Rio Grande do Sul, Brazil; 6 Laboratório de Pesquisa em Micobactérias, Faculdade de Medicina, Universidade Federal de Minas Gerais, Belo Horizonte, Minas Gerais, Brazil; 7 Centro de Pesquisa em Tuberculose, Faculdade de Medicina Universidade Federal do Rio de Janeiro, Rio de Janeiro (RJ), Brazil

**Keywords:** Brazil, drug resistance, genotyping, tuberculosis (TB)

## Abstract

Tuberculosis (TB) is the leading cause of death among infectious diseases worldwide. Among the estimated cases of drug-resistant TB, approximately 60% occur in the BRICS countries (Brazil, Russia, India, China and South Africa). Among Brazilian states, primary and acquired multidrug-resistant TB (MDR-TB) rates were the highest in Rio Grande do Sul (RS). This study aimed to perform molecular characterisation of MDR-TB in the State of RS, a high-burden Brazilian state. We performed molecular characterisation of MDR-TB cases in RS, defined by drug susceptibility testing, using 131 *Mycobacterium tuberculosis (M.tb)* DNA samples from the Central Laboratory. We carried out MIRU-VNTR 24loci, spoligotyping, sequencing of the *katG*, *inh*A and *rpo*B genes and RD^Rio^ sublineage identification. The most frequent families found were LAM (65.6%) and Haarlem (22.1%). RD^Rio^ deletion was observed in 42 (32%) of the *M.tb* isolates. Among MDR-TB cases, eight (6.1%) did not present mutations in the studied genes. In 116 (88.5%) *M.tb* isolates, we found mutations associated with rifampicin (RIF) resistance in *rpo*B gene, and in 112 isolates (85.5%), we observed mutations related to isoniazid resistance in *kat*G and *inh*A genes. An insertion of 12 nucleotides (CCAGAACAACCC) at the 516 codon in the *rpo*B gene, possibly responsible for a decreased interaction of RIF and RNA polymerase, was found in 19/131 of the isolates, belonging mostly to LAM and Haarlem families. These results enable a better understanding of the dynamics of transmission and evolution of MDR-TB in the region.

## Introduction

Globally, tuberculosis (TB) is the leading cause of death among infectious diseases, given that 10 million people became ill in 2017. An estimated 1.6 million people died because of the disease (including 300 000 among people with HIV). Drug-resistant TB (DR-TB) is a continuing threat with 558 000 new cases with resistance to rifampicin (RIF), the most effective first-line drug, of which 82% had multidrug-resistant TB (MDR-TB), and only 160 684 DR-TB were detected. Among the estimated cases of DR-TB, approximately 60% occur in the BRICS countries (Brazil, Russia, India, China and South Africa) [1].

The management of MDR-TB is characterised by delayed diagnosis, uncertainty of the extent of bacillary drug resistance, imprecise standardised drug regimens and dosages, very long duration of therapy and high frequency of adverse events associated with a high morbidity and mortality [2]. Recently, in a meta-analysis of 74 studies, including 17 494 DR-TB participants [3], the pooled treatment success rate was 26% in extensively drug-resistant TB (XDR-TB) patients and 60% in MDR-TB patients. MDR-TB is characterised as a resistance to isoniazid (INH) and RIF, and XDR-TB is defined as a resistance to all first-line drugs plus at least one fluoroquinolones and second-line injectable (kanamycin, amikacin or capreomycin) [1].

The *rpo*B (RNA polymerase gene) accounts for 96% of RIF resistance. RIF monoresistance is rare and occurs mostly together with resistance to other drugs, usually INH, making RIF a good marker for MDR [4]. The most common INH resistant mutation is in *kat*G (catalase peroxidase gene), which accounts for 64.2% and *inh*A (enoyl-acyl reductase gene) with 19.2% of INH resistance [5].

Different molecular tools have been developed for *Mycobacterium tuberculosis* (*M.tb)* genotyping. Spoligotyping and Mycobacterial interspersed repetitive units of a variable number of tandem repeats (MIRU-VNTR) applied together have been used, generating satisfactory discriminatory power for strain lineage classification [6]. More recently, greater access to the drug sensitivity testing (DST) has been proposed through gene sequencing platforms aimed at clinical decision making (e.g. replacing phenotypic DST) [7]. These tools allow an epidemiological investigation of TB transmission and relationship between strains lineage [8].

Strain variation in *M.tb* complex can have different phenotypic consequences, such as difference in gene expression, growth rates and metabolic responses [9]. The genetic background of *M.tb* strain can also affect the outcome of the infection and the response to drug resistance [10]. A molecular understanding of MDR strains through the analysis of resistance-conferring mutations and identification of lineages of *M.tb* in each region, as well as its interaction with different scenarios, enables a better comprehension of the dynamics of the infection, thus helping the improvement of the TB surveillance worldwide, also contributing with data for new treatment approaches [11].

Rio Grande do Sul (RS), in the southern region of Brazil, is a high-burden state, currently in fourth place in TB incidence rate among the Brazilians states with 39.5/100 000 cases and MDR-TB cure rate of 51.5% [12]. Among Brazilian states, primary and acquired MDR-TB rates were highest in RS, at 2.2% and 12%, respectively [13]. Therefore, the objective of this study was to characterise MDR *M.tb* isolates from RS state through the analysis of *kat*G, *inh*A and *rpo*B genes, as well as RD^Rio^ sublineage identification and MIRU-VNTR 24loci and spoligotyping methods.

## Methods

### Study population and *M.tb* culture

The *M.tb i*solates were collected from sputum samples of patients treated at the Hospital Sanatório Paternon (HSP) (a reference centre for TB resistance treatment of the State), during the years of 2013 and 2014. In this period, 18-year-old or older eligible participants, presenting coughs for 3 weeks or more and those with DST performed and presenting bacteriological confirmation of TB, and at least, one of the following conditions for defining them as presumed DR/MDR-TB: (a) with previous anti-TB treatment: being suspected of re-treatment failure or treatment defaults; (b) without previous anti-TB treatment: being HIV-seropositive subjects, or close contact with smear-positive MDR-TB cases. Subjects were excluded if they presented: (a) confirmed drug-sensitive TB, (b) refused to sign the Informed Consent (c) or harboured atypical mycobacteria.

During 2013 and 2014, HSP notified, in the SITE-TB website (database of DR-TB cases reported in Brazil), 186 MDR-TB cases. These 186 drug-resistant samples were submitted to the DST using the liquid MGIT-960 SIRE kit (Becton Dickinson Diagnostic Systems, Sparks, MD, USA) to first-line anti-TB drugs; RIF (1.0 µg/ml), INH (0.1 µg/ml), ethambutol (EMB) (5.0 µg/ml) and streptomycin (STR) (1.0 µg/ml) at the State Central Laboratory (LACEN), the TB diagnosis reference laboratory of the RS State. In this study, 131 MDR *M.tb* isolates were included. The project was approved by the Research Ethics Committee of the Fundação Estadual de Produção e Pesquisa em Saúde (FEPPS/RS), protocol number 1.587.621 CAAE: 18269313.0.0000.5320.

### DNA extraction

The genomic DNA of *M.tb* was extracted from sputum culture in Lowenstein–Jensen solid medium using Cetyltrimethylammonium Bromide (CTAB) method, as described by van Embden *et al*. [14].

### Spoligotyping

Spoligotyping was performed using the Beamedex microsphere technique (Beamedex SAS, Orsay, France) in the Luminex-Bioplex-BioRad 200 system (Luminex Corporation, Austin, TX, USA), developed in the ‘Institut de Genétique et Microbiologi e Université Paris-Sud’, following the protocol described by Zhang *et al*. [15]. The definition of the family, lineage level and definition of the spoligotype international type were performed by comparison with profiles deposited in the SITVITWEB database (http://www.pasteur-guadeloupe.fr:8081/SITVIT_ONLINE); when we found unknown or orphan profiles in SITVITWEB, we used SpotClust database to classify the isolates (http://tbinsight.cs.rpi.edu/run_spotclust.html).

### MIRU-VNTR 24loci

MIRU-VNTR 24loci was performed as described by de Beer *et al*. [16]. Fragment size of the amplicons was analysed on an ABI 3130xl DNA sequence analyser (Applied Biosystems, Foster City, California, USA) and the number of copies of each locus was determined by automated assignment using the Genemapper 3.2.1 software (Applied Biosystems). Undefined results or locus that did not amplify were double checked on agarose gels comparing to a reference table described by Supply *et al*. [17].

Lineage identification of *M.tb* isolates was carried out by best match analysis and Tree-based identification tools on the MIRU-VNTR*plus* database (https://www.miru-vntrplus.org) [6]. We applied 0.3 maximum tolerance of difference of four loci, as recommended by the MIRU-VNTR*plus* website for secure classification [6].

The discriminatory power was determined by the Hunter–Gaston discriminatory index (HGDI) [18] calculated online by using the discriminatory power calculator tool available at http://insilico.ehu.es. The allelic diversity of each of the MIRU-VNTR 24loci was determined at the MIRU VNTR*plus*, classified into highly (HGDI > 0.6), moderately (HGDI > 0.3) or poorly discriminative (HGDI < 0.3). The recent transmission rate was estimated by the N − 1 method [19], according to the formula: number of clustered isolates − number of clusters/total number of isolates.

The classification of the isolates by MIRU-VNTR and spoligotyping was performed based on dendrogram provided in the MIRU-VNTR*plus* by the construction of a Neighbour-Joining based phylogenetic tree [6].

### *kat*G, *inh*A and *rpo*B sequencing

Sequencing of hotspot regions of genes *kat*G, *inh*A and *rpo*B was performed according to Dalla Costa *et al*. [20]. Mutation analysis was conducted using SeqScape^®^ and Chromas^®^ software, and also the online platform Blast (http://blast.ncbi.nlm.nih.gov).

### RD^Rio^

RD^Rio^ sub-lineage was performed using the protocol described by Lazzarini *et al*. [21] and analysed on 1.5% agarose gel.

## Results

### Patients' characteristics

Among the 131 MDR-TB cases included, 100 (76.3%) were male, 72 (55%) were in an age bracket of 26–45 years, 124 (94.7%) with pulmonary TB, 57 (43.5%) were current tobacco smokers, 54 (41.2%) were illicit drug users, 37 (28.2%) had alcoholism and 33 (25.1%) had AIDS. Ninety-one (69.5%) had TB in the past; among them, 35 (26.7%) with a history of failure (Table 1).

**Table 1.** Socio-demographic characteristics of 131 MDR TB patients

Characteristic	*N* (%)	Characteristic	*N* (%)
Gender		Education (years)	
Male	100 (76.3)	None	4 (3)
Female	31 (23.7)	1–3	20 (15.3)
Age (years)		4–7	56 (42.7)
15–17	2 (1.5)	8–11	12 (9.2)
18–25	18 (13.7)	Ignored	39 (29.8)
26–45	72 (55.0)	Criteria for DST	
⩾46	39 (29.8)	Previous treatment failure	35 (26.7)
Occupation		HIV co-infection	20 (15.3)
Self-employed/salaried employee	44 (33.6)	Contact with DR-TB	14 (10.7)
Prisoners	22 (16.8)	Defaulted initial treatment	13 (9.9)
Unemployed	16 (12.2)	Disease recurrence	13 (9.9)
Housewives	13 (9.9)	Prisoner	11 (8.4)
Homeless	6 (4.6)	Hospitalised	9 (6.9)
Retired	3 (2.3)	Treatment control	7 (5.3)
Professional at the prison system	1 (0.8)	Immunosuppressed	1 (0.8)
Student	1 (0.8)	Homeless	1 (0.8)
Others	25 (19)	Retreatment	1 (0.8)
		Not informed	6 (4.6)
Treatment outcome^a^			
Favourable	68 (51.9)		
Unfavourable	63 (48.1)		

MDR-TB, multidrug-resistant tuberculosis. DR-TB, drug-resistant tuberculosis.

a Favourable treatment outcome: cured patients and patients that completed anti-TB treatment. Unfavourable treatment: patients that abandoned or failure to anti-TB treatment, died by TB or other cause.

Seventeen patients (13%) were additionally resistant to STR, 13 (10%) resistant to EMB and four (3%) resistant to both STR and EMB. The criteria for submission to DST are described in Table 1. Anti-TB treatment outcome, favourable and unfavourable results were identified in 68 (51.9%) and 63 (48.1%), respectively (Table 1).

### Mutations

Even though all 131 *M.tb* isolates were considered MDR according to the DST, eight (6.1%) did not present mutations in the *kat*G, *inh*A or *rpo*B genes. In 116 (88.5%) *M.tb* isolates, we found mutations associated with RIF resistance (in the 511, 512, 513, 516, 526, 531 codons in the *rpo*B gene), and in 112 isolates (85.5%), we observed mutations related to INH resistance (in the 315 codon in the *kat*G gene and −15 nucleotide of the *inh*A promoter region) (Table 2). We also found an insertion of 12 nucleotides (CCAGAACAACCC) at the 516 codon in the *rpo*B gene in 19 (14.5%) isolates. The mutation −17 (T-G) in the *inh*A gene was not found.

**Table 2.** Genotypic profile of 131 *M.tb* isolates

Isolates (*N*)	Mutation in *inh*A	Mutation in *kat*G	Mutation in *rpo*B	%
38	WT	S315T	S531L	29
27	−15 C-T	S315T	S531L	20.6
14	WT	S315T	Insertion 12 bp (516–517)	10.7
8	WT	WT	WT	6.1
8	WT	WT	S531L	6.1
5	WT	S315T	WT	3.8
4	−15 C-T	WT	S531L	3.1
3	WT	S315T	H526Y	2.3
3	WT	S315T	H526D	2.3
2	WT	S315T	S512 T and D516Y	1.5
2	−15 C-T	S315T	WT	1.5
2	−15 C-T	S315T	Insertion 12 bp (516–517)	1.5
1	WT	S315N	H526Y	0.8
1	−15 C-T	WT	S531F-L (double peak)	0.8
1	WT	S315R	Q513E and D516 V	0.8
1	−15 C-T	WT	L511P	0.8
1	−15 C-T	WT	Insertion 12 bp (516–517)	0.8
1	WT	S315T	S531W	0.8
1	WT	S315T	Insertion 12 bp (516–517) and S531L	0.8
1	WT	S315N	H526D	0.8
1	−15 C-T	S315T	H526D	0.8
1	−15 C-T	S315T	H526N	0.8
1	WT	WT	H526N	0.8
1	WT	S315T	H526C	0.8
1	WT	WT	Insertion 12 bp (516–517)	0.8
1	−15 C-T	S315T	S531W	0.8
1	WT	WT	H526D	0.8

WT, wild type.

Codons change and legend: KatG S315T = AGC → ACC, katG S315R = AGC → AGA, katG S315T = AGC → ACA, katG S315N = AGC → AAC, rpoB L511P CTG-CCG, rpoB S512T AGC-ACC, rpoB Q513E CAA-GAA, rpoB D516Y = GAC → TAC, rpoB D516V = GAC → GTC, rpoB H526Y = CAC → TAC, rpoB H526D = CAC → GAC, rpoB H526C = CAC → TGC, rpoB H526N = CAC → AAC, rpoB S531L = TCG → TTG, rpoB S531W = TCG → TGG, rpoB S531F-L = TCG → TTC-TTG (double peak).

### Spoligotyping

With a clustering rate of 0.61 and a discriminatory power of HGDI of 0.68, the 131 *M.tb* isolates analysed by spoligotyping were classified as the following: 66 (50%) LAM family (LAM 1, LAM2, LAM3, LAM4, LAM5, LAM6, LAM7, LAM9), 23 (17.5%) T family (T1, T2, T5-Madrid), 20 (15.2%) H family (H1, H3), 13 (9.9%) PINI2 family, two (1.5%) X family (X2), two (1.5%) Family33, two (1.5%) Family34, one (0.7%) Family36, one (0.7%) Atypic family and one (0.7%) was classified as S family.

### MIRU-VNTR 24loci

In the analysis by MIRU-VNTR, the clustering rate was 0.13 and the discriminatory power of HGDI was 0.66. Out of the 131 isolates, we found 64 of them (48.8%) classified as LAM family, 24 (18.3%) Haarlem family, two (1.5%) X family, two (1.5%) H37RV family, one isolate each (0.8%) were Uganda family, S family, Cameroon family and Delhi/CAS family. It was not possible to classify the families of 35 of these isolates (26.7%) within the 0.3 relaxed value recommended by the MIRU-VNTR*plus* in the identification by similarity search, but it was possible through the tree-based identification in combination with the spoligotyping patterns (Supplementary S1).

Out of the 24 *loci*, the highest allelic diversity indices (*h* > 0.6) were for QUB-26, QUB-11b, Mtub04, MIRU 23, MIRU 40, MIRU 26 and Mtub21. The *locis* MIRU 02, MIRU 04, Mtub29, MIRU 39 and MIRU 24 presented low allelic diversity indices (*h* < 0.3) (Supplementary S2).

### MIRU-VNTR and spoligotyping combined analysis

A combined analysis of MIRU-VNTR and spoligotyping through the MIRU-VNTR*plus* was also conducted to better classify the isolates, the distribution of the lineage was: 86/131 (65.6%) belonging to the LAM family, 29/131 (22.1%) Haarlem family, 5/13 (3.8%) Uganda family, 4/131 (3%) were X family, 4/131 (3%) H37RV family and with 1/131 each (0.8%) belonged to the S family, TUR family and West African (Supplementary S1). The discriminatory power of HGDI was 0.52 and the recent transmission index related to the clustering rate was 6.8%.

### RD^Rio^

The deletion RD^Rio^ was present in 32% of our isolates (42/131), while 67.9% were WT (89/131). Among the isolates that presented the deletion, 40 of them were classified as LAM family, one was TUR and another one Uganda in the combined analysis of MIRU-VNTR and spoligotyping. The LAM sublineage classified through spoligotyping were LAM 5 (14 isolates), LAM 9 (nine isolates), LAM 1 (six isolates), LAM 4 (six isolates) and LAM 2 (three isolates).

We investigated the possible correlation between treatment outcome with the different *M.tb* lineages and treatment outcome with different profiles of *kat*G, *inh*A and *rpo*B mutations, using the logistic regression model. To analyse the possible association between the RD^Rio^ genotype and treatment outcome, we used the *χ*^2^ test. Considering the *P*-value < 0.05, no correlation was found in both statistical analyses.

## Discussion

RS is a high-burden state currently in fourth place in TB incidence rate among the Brazilians states, and the city of Porto Alegre is the capital with more TB retreatment cases registered (31.2% of all cases) in 2017 [12]. For a better understanding of the distribution of the MDR strains and the frequency of mutation-associated resistance in the State, we performed genotyping through MIRU-VNTR 24loci and spoligotyping of 131 MDR *M.tb* isolates, as well as sequencing of drug resistance mutations.

By sequencing the *kat*G gene, mutations in 106 of our isolates (80.9%) at 315 codons were observed, agreeing also with other studies from the same state [22] and other regions [4]. Regarding the *inh*A gene, 41 MDR *M.tb* isolates (31.3%) had mutation in the promoter region of the gene. The frequency of mutations related to the *inh*A gene varies from different places [23], even when compared to sampling from the same region [22]. In this study, we did not find mutation on −17 position, as described recently in the same state [22].

Out of the 26 isolates (19.8%) that did not show any mutation in the *kat*G gene, seven of them had mutation in the *inh*A promoter region, the other 19 (14.5%) presented no mutations in both genes. Furthermore, 90 isolates (68.7%) showed no mutation in the *inh*A gene, indicating a low mutation rate in this gene in accordance with Zhang and Yew [24]. Some studies associated the high frequency of *kat*G mutations with MDR isolates and *inh*A mutations with monoresistant isolates [25]. A possible explanation for this would be that mutations in the promoter region of the *inh*A region generate a greater biological cost to the bacillus, and the isolates with the mutation in the *kat*G gene would be selected to survive. Thus, the presence of mutation in *kat*G provides to the bacillus greater probability of *M.tb* isolate to evolve to MDR [25, 26]. Our findings corroborate this hypothesis, since we also found a high frequency of *kat*G mutations and low frequency of *inh*A mutations among our MDR *M.tb* isolates.

Different mechanisms contribute to drug resistance acquisition. Intrinsic mechanisms developed by *M.tb* throughout its evolution, such as cell wall permeability, efflux pumps and alteration and degradation of the drug, facilitate the effect of the drug to be neutralised [27]. Furthermore, mutations in intergenic regions, such as ahpC-oxyR in the INH resistance, may cause phenotypic resistance without the presence of commonly known mutations [23].

Our cumulative mutation rate in *kat*G and *inh*A was 85.5%, a global review carried out by Seifert *et al*. [23], which described a similar rate in several studies, affirms that at least 84% of *M.tb* isolates with INH phenotype resistance are detectable with molecular diagnosis, based on the analysis of these mutations. Besides the fact that these mutations are present in <0.1% of isolates susceptible to INH, the diagnosis through these mutations has a specificity above 99% as markers of phenotypic resistance to INH [23].

In the analysis of the *rpo*B gene, the most frequent mutations found were in the 531, 516 and 526 codons, the same as reported in other studies with RIF-resistant isolates [22, 28]. The mutation in the codon 531 stands out with higher frequency (61.8%), also found in other studies in the same state [22, 29], in other regions of Brazil [30, 31] and other countries [28]. The codon 516 in the *rpo*B gene was mutated in 16.8% of the isolates and 2.3% of them had single nucleotide exchange, other studies show a bigger mutation rate with SNP in this codon [30, 32]. The codon 526 was mutated in 9.9% of the isolates, and similar results were found in MDR isolates from a nearby state [31]. Moreover, we found 15 (11.4%) isolates WT for the *rpo*B gene, similar to that described by Perizzolo *et al*. [29] and De Freitas *et al*. [30] with samples from the same State.

Another important finding in codon 516 of *rpo*B was an insertion of 12 nucleotides (CCAGAACAACCC) that was described for the first time by Perizzolo *et al*. [29]. It was observed in 19/131 (14.5%) of our samples, and a similar frequency was also found by Esteves *et al*. [22] in a similar study in the same region of Brazil. According to Esteves *et al*. [22], the insertion was present only in isolates classified as LAM family, previously wrongly classified as PINI2 by spoligotyping. However, in this study, we found the insertion present mostly in LAM family (12/19), but also in Haarlem family (5/19), West African family (1/19) and another one in TUR family by the combined analysis of MIRU and spoligotyping. Among the isolates with the insertion, there were two clusters with two isolates each that belonged to bigger clusters (LAM and Haarlem family). We noticed that out of the 19 isolates with this insertion, five were prisoners and one was a worker at the prison system; even though it is not so representative, it could be a way of transmission of these strains.

A study *in silico* that has been carried out by our group [33] indicates that this insertion in codon 516, with a duplication of four amino acids, decreases the binding efficiency of RIF and RNA polymerase through a conformational change in a region close to the RIF binding region. Another study done by Malshetty *et al*. [34] with *Mycobacterium smegmatis* (*M. smegmatis*) also shows that an insertion is related to RIF resistance due to changes in spatial conformation in the *rpo*B amino acids. *M. smegmatis* and *M.tb* are very similar, and the *rpo*B gene is highly conserved among them, so mutations in *M. smegmatis* model have great relevance in *M.tb* studies [34]. There are still few studies describing this 12-nucleotide insertion in the *rpo*B gene, all that we could find until now were two studies with sampling originating from the same state of our study; therefore, more studies on this are necessary to clarify its importance in the acquisition and transmission of MDR isolates.

The deletion RD^Rio^ was present in 32% of our isolates (42/131), and a similar frequency (38%) was found in the same State [35] and other regions of Brazil [36]. In disagreement with the previous results that found RD^Rio^ exclusively in LAM Family [21, 36, 37], we found the deletion also in TUR and Uganda Family in the combined analysis of MIRU-VNTR and spoligotyping, and classified as LAM and T family, respectively, through spoligotyping alone. This finding is contrary to the initial hypothesis of Lazzarini *et al*. [21] in which the RD^Rio^ genotype would be a marker exclusive to LAM sublineage. However, TUR and Uganda family can be a result of convergent evolution and homoplasy, since the molecular markers used in these methodologies can change and emerge into different strains [6, 9, 38, 39].

Some studies associate RD^Rio^ deletion with an increased transmissibility of the bacillus [40], treatment failure [41], drug resistance [42] and increased number of pulmonary cavitation [43], associating RD^Rio^ with a more severe form of TB. However, there are studies that did not find associations between RD^Rio^ strains and unfavourable clinical outcome [36]. Therefore, further investigation is necessary to understand the relationship between the clinical complications and the RD^Rio^ genotype, mainly through X-ray analysis, qualitative clinical data and patient follow-ups.

In the present study, the combined genotype methods (MIRU and spoligotyping) presented LAM family as 65.6% and Haarlem family 22.1% of our isolates, in accordance with other studies in the same state [22, 29, 35] and other regions of Brazil [30, 44]. Most of these studies also show T family as one of the most frequent among Brazilian *M.tb* strains. In our analysis by spoligotyping alone, we also observed T family as the second most frequent (17.5%), but when combined with MIRU-VNTR, these families were mostly classified as Haarlem and LAM lineage, increasing the number of these genotypes. The same happens observing the isolates classified as PINI2 family by spoligotyping, but identified as LAM genotype in the combined analysis, confirming the study of Dalla Costa *et al*. [45]. Differences in classification through MIRU-VNTR and spoligotyping were first described in isolates from Brazil by Vasconcellos *et al*. [46] and are in accordance with our study.

The higher frequency of LAM family in the Brazilian territory can be explained by the Portuguese colonisation in the 15th and 16th centuries, since Portugal also shows high frequency of LAM isolates [47], as well as in African countries such as Mozambique, which also was involved in migratory flow to Brazil in the 16th century [48]. Different *M.tb* lineages are associated with specific regions, different human population and different ethnic groups [38, 49], thus the human migration process contributed on shaping the current scenario of *M.tb* population globally [50].

Some isolates were classified as unknown by MIRU method alone; however, we obtained an identification when we analysed it together with spoligotyping. Regarding the clustering rate, the MIRU-VNTR 24loci rate was 0.13, while spoligotyping was 0.61, showing the importance of the combined analysis for a better classification of the isolates.

About the clustering analysis, 118 *M.tb* isolates had unique patterns and 13 were grouped in four clusters. Out of these clusters, three had three isolates each and one cluster had four isolates. It was also noticed that in almost every cluster, there were isolates with the 12 nucleotide insertion in the *rpo*B gene. The presence of clusters with identical genotypes among the isolates is an indicator of the transmission of the same strain [51]. In this study, no correlation about the clusters and patient's information was found to determine any kind of transmission, probably due to low clustering rate of the isolates. Our low recent transmission rate (6.8%), determined by the clusters in relation to the total number of isolates, is possibly due to the short time of sampling, same limitation found by Xu *et al*. [52]. The comparison of recent transmission rate is a valuable information to monitor strain transmission over the years [53].

By analysing the patient characteristics of this study, we observed that the majority of the patients was self-employed/salaried employee followed by prisoners and unemployed, with years of schooling from 4 to 7, who had previous treatment failure, HIV co-infection, besides other comorbidities, such as tobacco smoking and consumption of illicit drugs. These show the characteristics of a vulnerable population with a weakened immune system, which favours the acquisition of MDR-TB [54]. Although studies show that some families are related to some worsening in TB treatment and transmission [55, 56], in this study no correlations were observed regarding the patients' characteristics and a specific family, or regarding treatment outcomes and the families.

This study contributes valuable information about the molecular characterisation of MDR strains of the Southern Brazilian population. It helps to understand the transmission dynamics of the period, as well as collaborates with epidemiological data of the most frequent genotypes in the State of RS, thus contributing with the monitoring of MDR-TB strains in the region and the Country.

## Supplementary material

For supplementary material accompanying this paper visit https://doi.org/10.1017/S0950268819001006. click here to view supplementary material
