# Supplementary figures and images for: Molecular characterisation of multidrug-resistant Mycobacterium tuberculosis isolates from a high-burden tuberculosis state in Brazil
Source: Epidemiol Infect. 2019 Jun 13;147:e216. doi: 10.1017/S0950268819001006 (PMC6624858; doi:10.1017/S0950268819001006)

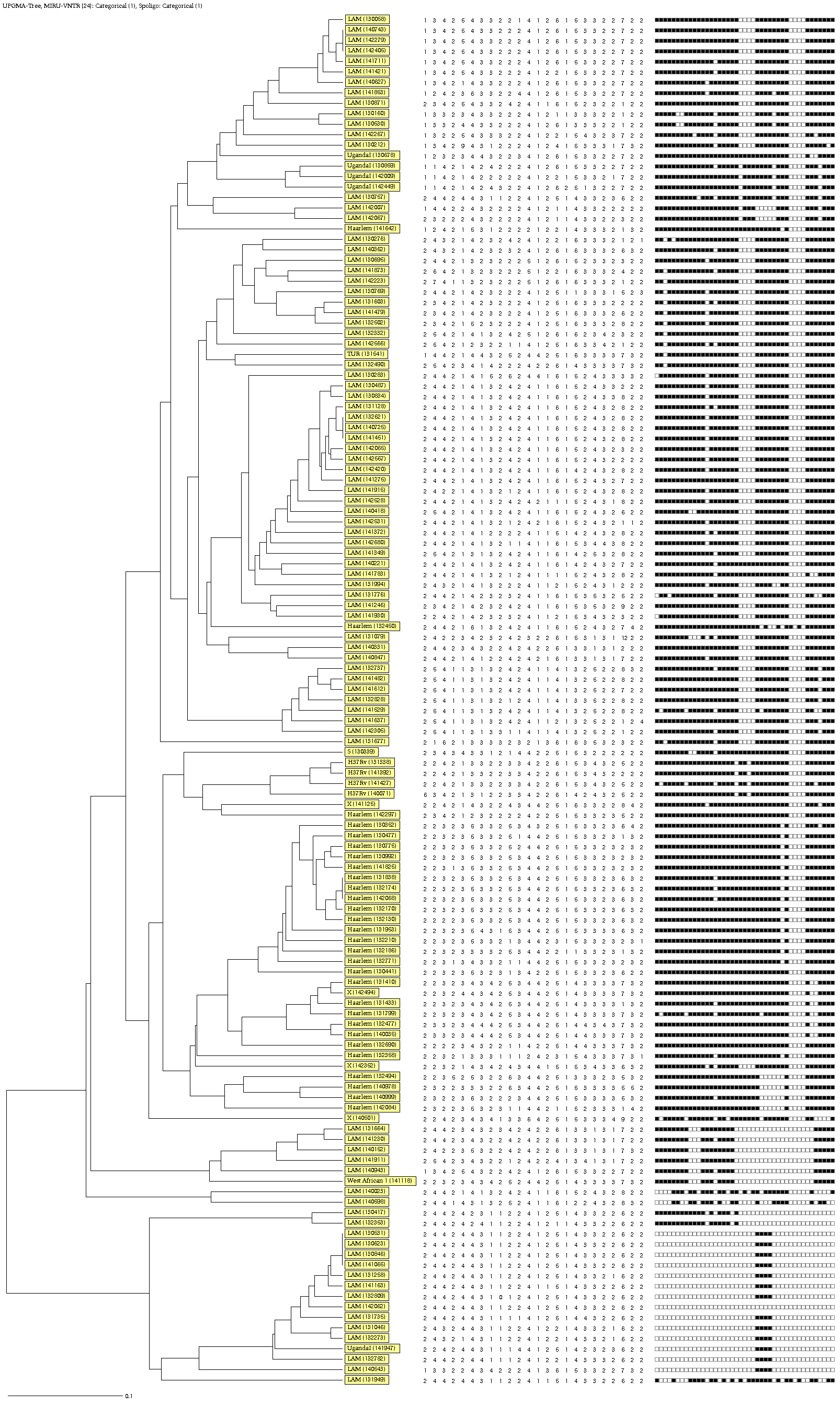

Supplement: Supplementary file 1 [file S0950268819001006sup001.png]
